# *Ctenocephalides felis* (Siphonaptera, Pulicidae) parasitizing White-eared opossum (*Didelphis albiventris*) at Southern Brazil - Case report

**DOI:** 10.29374/2527-2179.bjvm0042223

**Published:** 2023-12-04

**Authors:** Julia Somavilla Lignon, Diego Moscarelli Pinto, Silvia Gonzalez Monteiro, Gabrielle Torres Cotta de Mello, Natália Soares Martins, Tamires Silva dos Santos, Felipe Geraldo Pappen, Fábio Raphael Pascoti Bruhn

**Affiliations:** 1 Veterinarian, Laboratório de Epidemiologia Veterinária, Departamento de Veterinária Preventiva, Universidade Federal de Pelotas, Pelotas, RS, Brazil; 2 Veterinarian, Laboratório do Grupo de Estudos em Enfermidades Parasitárias, Departamento de Veterinária Preventiva, Universidade Federal de Pelotas, Pelotas, RS, Brazil; 3 Veterinarian, Laboratório de Parasitologia Veterinária, Departamento de Microbiologia e Parasitologia, Universidade Federal de Santa Maria, Santa Maria, RS, Brazil

**Keywords:** ectoparasites, fleas, saruê, marsupial, zoonosis, ectoparasitos, pulgas, saruê, marsupial, zoonose

## Abstract

*Didelphis albiventris* is a marsupial with synanthropic habits, whose contact with domestic animals and humans is increasing. They are considered potential disseminators of diseases, present as definitive hosts or reservoirs of various pathogenic agents. The present study aim to report, for the first time, the parasitism by *Ctenocephalides felis* in *D. albiventris* in Rio Grande, Southern Rio Grande do Sul state, Brazil. An adult female White-eared Opossum (*D. albiventris*), killed by being run over, was collected on the roads of the city of Rio Grande, Rio Grande do Sul, Brazil and sent to the laboratory for necropsy. During external inspection, a flea was found, which was stored in 70^o^ alcohol until taxonomic identification, being classified as *C. felis*. This study reports the occurrence of parasitism by *C. felis* in *D. albiventris* for the first time in the location studied, demonstrating that these arthropods circulate between the wild and urban environments, and play an important role in the cycle of zoonotic pathogens among wild fauna, companion animals and humans.

## Resumo

*Didelphis albiventris* é um marsupial de hábitos sinantrópicos, cujo contato com animais domésticos e humanos está aumentando. São considerados potenciais disseminadores de doenças, apresentando-se como hospedeiros definitivos ou reservatórios de vários agentes patogênicos. O presente estudo tem como objetivo relatar, pela primeira vez, o parasitismo por *Ctenocephalides felis* em *D. albiventris* em Rio Grande, Sul do Rio Grande do Sul, Brasil. Uma fêmea adulta de gambá-de-orelha-branca (*D. albiventris*), morta por atropelamento, foi recolhida nas estradas da cidade de Rio Grande, Rio Grande do Sul, Brasil e encaminhado ao laboratório para necropsia. Durante inspeção externa foi encontrada uma pulga que foi armazenada em álcool 70º até a identificação taxonômica sendo classificado como *C. felis*. Este estudo relata a ocorrência de parasitismo por *C. felis* em *D. albiventris* para o primeira vez no local estudado, demonstrando que esses artrópodes circulam entre a natureza e ambientes urbanos e desempenham um papel importante no ciclo de patógenos zoonóticos entre a fauna selvagem, animais de companhia e humanos.

## Introduction

The white-eared opossum (*Didelphis albiventris* Lund, 1840) is a species of marsupial widely distributed in the Neotropical Region, covering countries such as Argentina, Bolivia, Brazil, Paraguay and Uruguay (Lemos & Cerqueira, 2002). Of nocturnal and crepuscular habits, they are terrestrial, omnivorous and opportunistic animals, and, therefore, these marsupials are well adapted to different environments, presenting high synanthropism, where contact with domestic animals and humans in rural areas, and even urban areas, is increasingly frequent (De Sá et al., 2014; Muller et al., 2005).

According to Bezerra-Santos et al. (2020), *D. albiventris* are considered potential disseminators of diseases among wild and domestic fauna and humans, since they act as definitive hosts or reservoirs of various pathogenic agents (i.e., protozoa, helminths, arthropods and rickettsiae).

Among arthropods, *Ctenocephalides felis* (Bouché, 1835), the most frequent flea on dogs and cats (Durden & Hinkle, 2019; Monteiro, 2017), has been attracting the attention of researchers. These insects are intermediate hosts of tapeworms, such as *Dipylidium caninum* and *Hymenolepis* spp. They can also transmit the nematodes *Dipetalonema reconditum* and *Dirofilaria immitis*, in addition to *Rickettsia felis*, a species recognized as pathogenic, which is part of the spotted fever group rickettsiae (Horta et al., 2005; Monteiro, 2017). *C. felis* has still been the subject of research due to its probable participation in the epidemiology of Canine Visceral Leishmaniasis (Coutinho & Linardi, 2007; Ferreira et al., 2009), whose opossums are considered reservoirs and are important in maintaining *Leishmania* in the environment (Organização Pan-Americana da Saúde, 2021).

Therefore, the objective was to report, for the first time, parasitism by *C. felis* in *D. albiventris* in Rio Grande, south of the state of Rio Grande do Sul, Brazil.

## Case report

An adult female White-eared Opossum (*D. albiventris*), killed by being run over, was collected on a road in the municipality of Rio Grande, Rio Grande do Sul estate (RS), Brazil (33º1'56''S; 52º51'19' 'O). The animal had preserved and unexposed viscera, with an estimated time of death between one and seven hours. The collected animal was placed in a plastic bag, labeled with species, sex, date, city and place (geographical coordinates) where it was found, and transported in an isothermal box with ice to the laboratory of the Grupo de Estudos em Enfermidades Parasitárias, Faculdade de Veterinária, from the Universidade Federal de Pelotas (UFPel), located in Capão do Leão/RS. In the laboratory, he was necropsied and during the external inspection a flea was found. The ectoparasite was stored in a 2mL microtube containing 70% alcohol. Subsequently, it was clarified in phenol/xylene according to the Costa Lima method (Hoffmann, 1987) with modifications, and mounted in a non-permanent preparation between slide and coverslip. The specimen was examined under optical microscopy (10x and 40x objective) and taxonomically classified as *C. felis*, female adult, as described by Monteiro (2017). The collection and transport of roadkill wild animals were authorized by the Authorization and Information System on Biodiversity of the Ministry of the Environment under registration 82632-3 based on Normative Instruction No. 03/2014. This work was also approved by the UFPel Ethics in the Use of Animals Committee (process number 23110.046990/2022-02).

## Discussion

**Figure 1:**
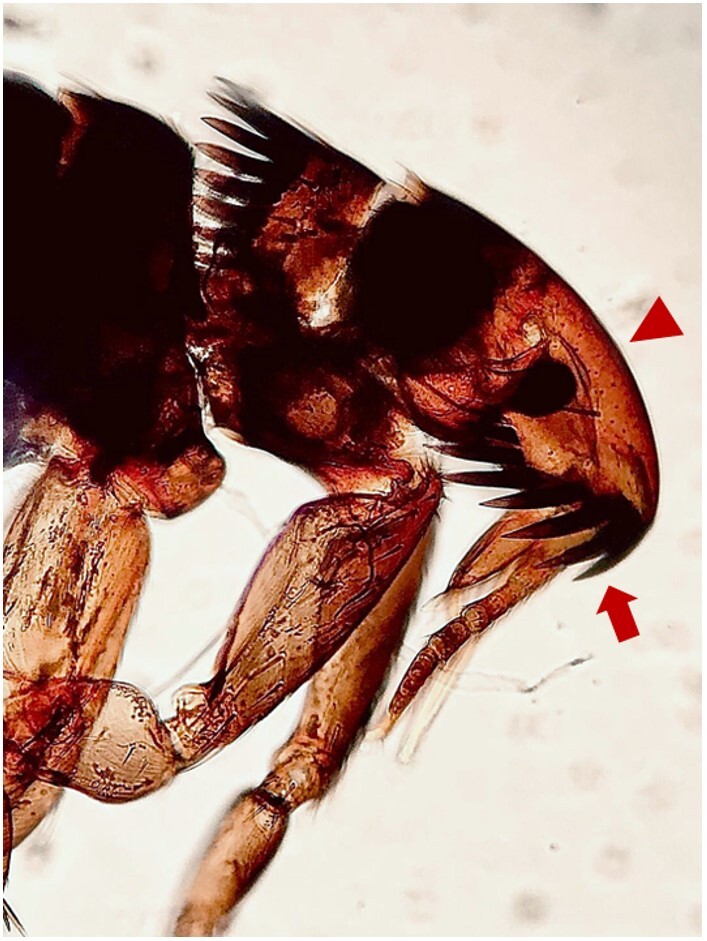
*Ctenocephalides felis* found parasitizing *Didelphis albiventris* in Rio Grande, RS, Brazil. It has a head with an oblique forehead (arrowhead) and eight segments of the genal ctenidium (arrow). 400x magnification.

The flea found was classified as *C. felis*, whose characteristics include a head with an oblique forehead, eight segments of the genal ctenidia, the first of which may be a little smaller than the others or the same size (Figure 1), in addition to the presence of spermatheca, indicating that it is an adult female flea (Monteiro, 2017).

*Ctenocephalides felis* was described parasitizing *D. albiventris* for the first and only time in RS in 2005, from the municipality of Pelotas and surroundings (Brum, 2005). Although it has already been described in other Brazilian states in this same host species (Barros-Battesti & Arzua, 1997) and in other species of *Didelphis* (Bezerra-Santos et al., 2020), almost 20 years have passed since the first description in RS and still little is known about ectoparasitic siphonapterans of *D. albiventris* in the state, and this is, so far, the first record of this parasitism in Rio Grande/RS.

*Didelphis albiventris* are possibly the most synatropic animals in the state of RS and as mentioned previously, *C. felis* can be an intermediate host and transmit several endoparasites, from urban to wild environments and vice versa. *R. felis* is an emerging pathogen that infects humans and is the causative agent of the probably underestimated flea-borne Rocky Mountain spotted fever (Peniche-Lara et al., 2016). This agent has been reported in different countries around the world infecting its biological vector (*C. felis*) and this species found in dogs has already been diagnosed with *R. felis* in the state of São Paulo, Brazil (Horta et al., 2005). Opossums (family Didelphidae) have also been found infected with *R. felis* in some countries in the Americas (Labruna et al., 2007, 2011; Peniche-Lara et al., 2016). Specimens of *D. virginiana* have been implicated in the zoonotic transmission of *R. felis* in the United States (Labruna et al., 2011), as well as *D. marsupialis* in Mexico (Peniche-Lara et al., 2016). These studies suggest that opossums can act in the horizontal and vertical transmission of these pathogens. Being sources of *R. felis* infection for uninfected vectors or as carriers of infected vectors to other mammalian hosts, such as domestic animals or companion animals, humans and wild mammals (Peniche-Lara et al., 2016). Although disease caused by *R. felis* in humans is considered less severe (no lethal cases reported) than disease caused by *R. rickettsii*, a differential diagnosis of both agents should be performed in areas where both agents exist. Furthermore, *R. felis* is a relatively new agent and many characteristic clinical and ecological effects have yet to be explored and elucidated (Horta et al., 2005).

Ferreira et al. (2009) also suggest the participation of *C. felis* fleas in the cycle of infection by *Leishmania*, a protozoan whose potential wild reservoir is the opossum. In a nearby municipality, belonging to the region of the present study, Macedo et al. (2019) found a prevalence of 34% (17/50) of this protozoan in *D. albiventris*, which was also described in this same host in other states of the country (Humberg et al., 2012; Quintal et al., 2011). Therefore, the importance of this report is emphasized, suggesting that more epidemiological studies should be carried out to elucidate these factors, since there are still no reports of the presence of the main vector (phlebotomine) responsible for the transmission of *Leishmania* in the region. The magnitude of the health problem of leishmaniasis and its complex epidemiology point to the need to identify all the links in the transmission chain, to implement effective control strategies. Thus, understanding each focus of transmission is essential to support effective and sustainable strategies for the surveillance of leishmaniasis.

The implication of opossums infested by *C. felis* resides in the very close association of these marsupials with human habitations (domestic and peridomestic), allowing them to potentially contribute to the transmission cycle of vector-borne zoonotic pathogens, narrowing the possibility of human infection. Therefore, the occurrence of this siphonapteran infesting *D. albiventris* in the southern region of RS, is an important epidemiological factor in this locality.

## Conclusion

This study reports the occurrence of parasitism by *C. felis* in *D. albiventris* for the first time in the municipality of Rio Grande, Rio Grande do Sul state, Brazil, demonstrating that these arthropods circulate between the wild and urban environments, and play an important role in the cycle of zoonotic pathogens among wild fauna, companion animals and humans.
